# Characterization and Genome Sequence of *Mycobacterium* Phage XianYue

**DOI:** 10.1128/MRA.00459-20

**Published:** 2020-06-18

**Authors:** Wesley D. Rhinehart, Amanda J. Laidlaw, Alexis M. O’Neal, Jessica A. Toller, Miriam Segura-Totten, Ryan A. Shanks, Alison E. Kanak

**Affiliations:** a Department of Biology, University of North Georgia, Dahlonega, Georgia, USA; Queens College

## Abstract

Novel mycobacteriophage XianYue was isolated in Northeast Georgia and infects Mycobacteria smegmatis mc^2^155. Actinobacteriophages which share at least 50% nucleotide identity are grouped into clusters, with XianYue in cluster A2. Its genome is 52,907 bp with 91 open reading frames (ORFs) and 62.9% GC content, and it shares 86.51% nucleotide identity with mycobacteriophage Trixie.

## ANNOUNCEMENT

Bacteriophage are abundant, with an estimated 10^31^ particles on Earth (1). Phage are studied in order to elucidate mechanisms of microbial evolution as well as a potential solution for antibiotic resistance in bacteria. Actinobacteriophages which share at least 50% nucleotide identity are arranged into clusters, with further categorization into subclusters where appropriate (2). In this paper, we describe the mycobacteriophage XianYue, isolated using a Science Education Alliance–Phage Hunters Advancing Genomics and Evolutionary Science (SEA-PHAGES) protocol (3).

XianYue was isolated from soil collected in Hart County, Georgia (34.386N, 82.949W). Mycobacterium smegmatis mc^2^155 was used as the host for XianYue isolation and characterization. Briefly, 7H9 liquid medium was added to soil and incubated at 37°C for 24 h. The sample was then filtered using a 0.22-μm filter. Phage was confirmed and purified via plaque assay and then amplified to a high titer to extract phage genomic DNA for sequencing. XianYue’s capsid measures 61 nm in diameter, and the tail is 128 nm long. It creates plaques 8 mm in diameter with distinct borders (4).

Phage genomic DNA was extracted from XianYue lysates with a Wizard DNA extraction kit (Promega). A NEBNext Ultra II FS kit with dual-indexed barcoding was used to assemble a sequencing library. Libraries from 47 other phages, in addition to XianYue, were gathered and run on an Illumina MiSeq system. There were ∼173,861 single-end 150-bp reads from the XianYue library that, when assembled, provided ∼454-fold coverage of the XianYue genome. These raw reads were assembled using Newbler v2.9 with default settings. The resulting single phage contig was checked for completeness, accuracy, and phage genomic termini using Consed v29 (5) as previously described.

The genome was annotated using GeneMark v3.25 (6), NCBI BLAST v2.9.0 (7), Glimmer v3.02 (8), HHpred v3.2.0 (9), ARAGORN v1.2.38 (10), and Phamerator (https://phamerator.org). Default parameters were used for all software unless otherwise specified. Hits with an E value of 10e^−4^ or less were considered acceptable. Phamerator and GeneMark indicate that XianYue has 93 open reading frames (ORFs), and 43 have predicted functions. Genes 1 to 34 and 36 are transcribed in the forward direction, while genes 35 and 37 to 91 are transcribed in the reverse direction. Like other A2 phages, XianYue is predicted to be temperate, as a tyrosine integrase (ORF 35) and immunity repressor (ORF 71) were identified. Of interest, ORF 36, immediately downstream of ORF 35, is predicted to code for a protein of unknown function and is transcribed in the opposite direction of the genes flanking it, a common feature of prokaryote transcriptional regulators (11–14). The structural genes, including the lysis cassette, of XianYue run from ORFs 6 to 31, while the right arm consists mainly of ORFs with unknown function, also typical for A2 phages. XianYue has 3′ sticky overhang genomic ends, with a 10-bp overhang. ORFs 24 and 25 overlap with a −1 frameshift and are predicted to be the tail assembly chaperones, which is frequently observed in A2 phages (15). XianYue is most genetically similar to Trixie (GenBank accession no. NC_023731.1), with 86.51% nucleotide identity via BLAST alignment.

### Data availability.

Information on XianYue’s genome can be found in GenBank under the accession no. MK814748. Sequencing reads are part of the Sequence Read Archive with accession no. SRX7260346 under BioProject accession no. PRJNA488469.
